# A New Medicinal Vegetable

**Published:** 1844-10

**Authors:** 


### A NEW MEDICINAL VEGETABLE.

A curious instance of the *crossing of plants*, by which a *hybrid* was produced, which promises to be a valuable addition to the Materia Medica, was mentioned to us a few weeks since, by Mr. Babbit, of Union Village, in Warren county, Ohio. This gentleman informed us that having procured some seed from the dried pulp of the *cucumis colocynthis* of the shops, he was curious to try whether they would vegetate, and accordingly planted them in the neighborhood of some water-melon seed. They grew and produced the genuine fruit of the colocynth. The water-melons were excellent, and the seed of a number of them were saved for future planting; but next year, instead of water-melons, Mr. Babbit was surprised to find growing on the vines from this seed a *hybrid* resembling more in appearance the colocynth than the melon, having a shell capable of holding about three pints, and growing yellow, like the colocynth, as it ripened. The taste of the pulp was found on trial to be bitter, and on making an extract from it, this turned out to possess the cathartic properties of the colocynth. Mr. B. placed some of the extract in the hands of the physicians of Lebanon, who esteem it as equal to the genuine article in activity. In Union Village, Mr. B. informed us, that he had enough of the fruit growing this year from the seed of the hybrid, to make sixty pounds of the extract. No change has taken place in the fruit since the first year, the seed of the new plant having continued to produce a melon of the same appearance and qualities. Mr. Babbit has politely promised to furnish us with some of the seed of his new vegetable, and as he has an abundant supply, we doubt not it would afford him pleasure to furnish others with it also. Y.

